# Evaluation of the Safety of Imatinib Mesylate in 200 Iraqi Patients with Chronic Myeloid Leukemia in the Chronic Phase: Single-Center Study

**DOI:** 10.4274/Tjh.2012.0135

**Published:** 2013-12-05

**Authors:** Bassam Francis Matti, Alaa Fadhil Alwan, Alaa Fadhil Alwan

**Affiliations:** 1 Baghdad Teaching Hospital, Clinical Hematology Department, Baghdad, Iraq; 2 National Center of Hematology, Clinical Hematology Department, Baghdad, Iraq

**Keywords:** Safety, Imatinib, Chronic myeloid leukemia

## Abstract

**Objective:** Imatinib mesylate, a tyrosine kinase inhibitor, is presently the drug of choice for chronic myeloid leukemia (CML). During therapy, a few patients may develop hematological and non-hematological adverse effects.

**Materials and Methods:** The aim of this study was to evaluate the safety of imatinib therapy in patients with CML. Between December 2007 and October 2009 two hundred patients with CML in chronic phase were included in the study. Written informed consent was obtained from all patients prior to the start of the study. Imatinib was started at 400 mg orally daily. Patients were monitored carefully for any adverse effects. Complete blood count, liver, and renal function tests were done once in 2 weeks during the first month and on a monthly basis during follow-up. Toxicities that encountered were graded as per the National Cancer Institute common toxicity criteria version 2. Both hematologic and non-hematologic toxicities were managed with short interruptions of treatment and supportive measures, but the daily dose of imatinib was not reduced below 300 mg/day.

**Results:** Two hundred CML patients in chronic phase were included in this study; the male:female ratio was 0.7:1 with mean age 39.06±13.21 years (ranged from 15-81 years). The study showed that the commonest hematological side effects were grade 2 anemia (12.5%) followed by leukopenia (8%) and thrombocytopenia (4%), while the most common non-hematological adverse effects were superficial edema and weight gain (51.5%), followed by musculoskeletal pain (35.5%), then gastro-intestinal symptoms (vomiting, diarrhea) (19%). Fluid retention was the commonest side effect, which responded to low-dose diuretics. The drug was safe and well tolerated. There were no deaths due to toxicity.

**Conclusion:** Imatinib mesylate a well-tolerated drug, and all undesirable effects could be ameliorated easily. The most common hematological and non-hematological side effects were anemia and fluid retention, respectively

**Conflict of interest:**None declared.

## Abstract (tr)

**Amaç:** Bir tirozin kinaz inhibitörü olan imatinib mesilat, günümüz kronik miyeloid lösemi (KML) itedavisinde ilk seçenek ilaçtır. Tedavi sırasında az sayıda hastada hematolojik ve non-hematolojik yan etkiler gelişebilir.

**Gereç ve Yöntemler:** Bu çalışmanın amacı KML hastalarında imatinib tedavisinin güvenirliğini değerlendirmektir. Aralık 2007-Ekim 2009 arasında kronik faz KML tanılı 200 hasta çalışmaya alındı. Çalışmanın başlamasından önce tüm hastalardan yazılı onam alındı. İmatinib oral yoldan günde 400 mg başlandı. Hastalar yan etkiler yönünden dikkatle izlendi. Tam kan sayımı, karaciğer ve böbrek fonksiyon testleri ilk ay içinde iki haftada bir, sonrasındaki takipte ise ayda bir yapıldı. İzlenen toksisiteler ulusal kanser enstitüsü toksisite kriterleri 2. versiyona göre derecelendirdi. Hematolojik ve non-hematolojik toksisiteler tedavinin kısa süre kesilmesi ve destek önlemleri ile yönetildi, ancak imatinibin günlük dozu 300 mg’ın altına inilmedi.

**Bulgular:** Çalışmaya 200 hasta dahil edildi. Erkek/kadın oranı 0,7:1, ortanca yaş 39,06 (15-81) idi. Çalışmada en sık rastlanan hematolojik yan etkiler grade 2 anemi (%12,5), lökopeni (%8) ve trombositopeni (%4) iken, en sık görülen non-hematolojik toksisiteleri yüzeyel ödem ve kilo alma (%51,5) takiben kas-iskelet ağrısı (%35,5) ve sonrasında gastrointestinal semptomlar (kusma, ishal-%19) olarak bulundu. En sık görülen yan etki düşük doz diüretiklere yanıt veren sıvı retansiyonuydu. İlaç güvenilir bulundu ve iyi tolere edildi. Toksisiteye bağlı ölüm izlenmedi.

**Sonuç:** İmatinib mesilat iyi tolere edilen bir ilaçtır ve tüm yan etkiler kolay yönetilebilir. En sık gözlenen hematolojik yan etki anemi, non-hematolojik yan etki ise sıvı retansiyonuydur.

## INTRODUCTION

Chronic myeloid leukemia (CML) arises as the result of a mutation in a pluripotent stem cell and is characterized by progressive granulocytosis, marrow hypercellularity, and splenomegaly [1,2,3]. CML accounts for about 20% of newly diagnosed cases of leukemia in adults [2,4]. The diagnostic hallmark is the Philadelphia chromosome [5], which is present in all dividing cells of hematopoietic lineage, as well as in B and T cells in some patients, but is absent in all other cells. The essential role of BCR-ABL tyrosine kinase activity for cellular transformation provides the rationale for targeting this function therapeutically [6].

Imatinib selectively inhibits the proliferation and induces apoptosis in BCR-ABL–positive cell lines as well as fresh leukemic cells from patients with Philadelphia chromosome-positive CML and Philadelphia chromosome-positive acute lymphoblastic leukemia [7,8]. Growth inhibition of the CML cell line K562 occurred at micromolar concentrations and was associated with inhibition of BCR-ABL tyrosine kinase activity [9]. In addition to that, imatinib inhibits the receptor tyrosine kinases for platelet-derived growth factors (PDGFs), stem cell factor (SCF), and c-kit and inhibit PDGF receptor and SCF-mediated cellular events [10].

The prospective International Randomized Imatinib Study (IRIS) showed clear superiority for imatinib when compared to interferon and low-dose cytarabine as standard therapy for CML. After a median follow-up of 19 months, the estimated rate of major cytogenetic response was 87.1% in the imatinib group and 34.7% in the interferon group. In regard to the molecular responses to imatinib mesylate, among 1106 patients from the IRIS study, 370 patients in complete cytogenetic response (CCR) were monitored by real-time quantitative polymerase chain reaction. Those who achieved a 3-log reduction from the initial BCR-ABL/BCR ratio after 12 months of therapy had a progression-free survival of 100% in 14 months, compared to 95% for those who had not achieved a 3-log reduction but were in CCR and 85% for those who had not achieved CCR at 12 months (p<0.001) [11].

Imatinib mesylate is a well-tolerated agent. In phase II trials with this drug, grade 3 to 4 hematologic toxicity was seen in 34% of chronic phase, 58% of accelerated phase, and 63% of blastic phase patients. Non-hematologic toxicity most commonly included nausea (58%-71%), fluid retention (56%-71%), muscle cramps (37%-50%), diarrhea (37%-53%), and skin rash (39%-43%) [12]. However, these adverse events were mostly mild and only rarely resulted in permanent discontinuation of therapy [13,14,15].

The updated 5-year IRIS study results showed that the rate of toxicity with first-line imatinib declined with time with; most of them being of grade 1 (mild) or 2 (moderate) in severity, generally able to be managed and tending to be most frequent in the first year of treatment. Imatinib discontinuation due to drug-related adverse effects was less than 4%. Grade 3 or 4 non-hematologic toxicities include fatigue, depression, myalgia, arthralgia, and nausea. Hematologic grade 3-4 toxicities within the first 2 years were reported to be neutropenia, thrombocytopenia, anemia, and elevated liver enzymes at 3.7%, 1.5%, 1.8%, and 0.4%, respectively [16]. The aim of the present study was to evaluate the safety of imatinib mesylate in CML patients. 

## MATERIALS AND METHODS

The study was conducted from December 2007 through October 2009; during this period, 200 patients with CML in the chronic phase treated with imatinib mesylate were evaluated at the National Center of Hematology by history, clinical examination, and laboratory tests. 

Eligibility criteria included age of 15 years and older, Eastern Cooperative Oncology Group (ECOG) performance status of 0 to 2, adequate hepatic and renal functions, no prior imatinib therapy, and absence of pregnancy. Chronic myeloid leukemia in the chronic phase was defined as less than 10% blasts and less than 20% basophils in the peripheral blood and bone marrow, and a platelet count of more than 100 x 109/L but less than 1000 x 109/L. The study was approved by the institutional ethics committee. Written informed consent was obtained from all patients prior to the start of the study. Therapy was initiated with imatinib at 400 mg orally daily and patients were monitored carefully for any adverse effects. Complete blood count and blood film, liver function tests, renal function tests, and coagulation parameters were recorded once in 2 weeks during the first month and monthly thereafter. Toxicities encountered were graded as per the National Cancer Institute’s common toxicity criteria, version 2. Both hematologic and non-hematologic toxicities were managed with short interruptions of treatment and supportive measures, but the daily dose of imatinib was not reduced below 300 mg/day. 

Regarding assessment of imatinib toxicity on the gastrointestinal tract, we assessed whether there was any nausea, vomiting, stomatitis, or diarrhea in patients who received imatinib. 

Any fever with or without infection in CML patients was evaluated by identifying any elevation in temperature at the time of registration and recording any history of fever, whether it was related to any infection or not. 

Dermatological abnormalities during the period of imatinib intake including any skin rash or itching, along with hair loss and color changes, were evaluated by direct examination. 

Neurological evaluation was done only by clinical neurological assessment with notification of any dysesthesia or paresthesia. 

For all patients, baseline weight was recorded and then weight measurements were done monthly after imatinib therapy began to determine any weight change. 

The statistical analysis was performed using SPSS 17.0 (SPSS Inc., Chicago, IL, USA). Differences between groups were evaluated by using Student’s t-test this statement should be omitted. P<0.05 was regarded as significant.

## RESULTS

In this study, 200 patients with CML in the chronic phase were included; 112 patients were female while 88 patients were male with a male-to-female ratio of 0.7:1. Age ranged from 15 to 81 years with a mean age of 39.06 ± 13.21 years; other pretreatment characteristics are shown in Table 1. Table 2 shows the hematologic and non-hematologic side effects, while Table 3 shows the distribution of side effects according to sex. 

## DISCUSSION

matinib has been generally well tolerated, with grade 3 or 4 toxicities being uncommon. The most common side effects of imatinib were usually of grade 1 or 2. 

The most common side effects encountered in this study were non-hematologic, with all grades of superficial edema (51.5%), followed by bone pain (35.5%) and then nausea (32.5%). 

Imatinib has the potential to induce severe and prolonged myelosuppression, particularly in patients with minimal residual normal hematopoiesis. However, over time, some patients can achieve recovery of normal hematopoiesis and a major cytogenetic response despite having experienced recurrent grade 3 neutropenia and thrombocytopenia and frequent dose interruptions [17,18]. 

Hematologic adverse effects included anemia, which was documented in 14% of CML cases, most of them of grade 1 or 2. It was found more frequently in females than in males (P = 0.0001); this may be due to the fact that the basal hemoglobin level is lower in females or that they are more sensitive to the myelosuppressive effect of imatinib than males. Grade 3-4 hematologic toxicity was registered only in 4.5%. Regarding the percentage of all grades, leukopenia and thrombocytopenia in this study were found at 10% and 4%, respectively, while grade 3-4 neutropenia was documented in 1%. Of all CML patients studied, 13.5% complained of hemorrhagic manifestations, all within the first few weeks of initiation of treatment. This may be due to concomitant platelet dysfunction, which is in agreement with the study by Druker et al., in which hemorrhage was experienced in 18.9% of patients. However, this study did not find that use of NSAIDs or other concomitant treatment significantly increased the risk of bleeding in CML patients [19]. 

Breccia et al. found that grade 3-4 hematologic toxicities were experienced in 24% of chronic phase CML patients; of these, 7% experienced toxicity of grades 3-4 in early chronic phase patients, with a negative influence on cytogenetic response [17]. 

Edema and fluid retention occurred in 51.5% of cases, manifested clinically by periorbital edema, leg edema, and generalized edema, which was significantly more common in female patients than in males (P<0.011). No ascites or anasarca cases were documented in this study; in addition, neither pleural nor pericardial effusions were registered in this study, similar to the findings of Breccia et al. [17]. Hensley et al. reported that the non-hematologic main adverse effects with imatinib included fatigue, edema, nausea, diarrhea, muscle cramps, and rash [18]; Druker et al. found similar results regarding fluid retention and edema at 53.2% [19], but severe periorbital edema was occasionally observed and was postulated to be an effect of platelet-derived growth factor receptor and KIT expressed by dermal dendrocytes [19,20]. 

Regarding bone pain, this study found that it was experienced by 35.5% of patients, which is similar to the rates reported by other studies [19,21]. 

Paresthesia was found in 15%, which was not proven by electromyography or nerve conduction study; this was highly significant in females (P<0.001), which may be due to hypophosphatemia, hypocalcemia, and hypomagnesemia caused either by the imatinib or NSAIDs. Low incidence of severe infections (grades 3 and 4) was noticed in 1.5% of patients with chronic phase CML, more significantly so in females. Typically patients with chronic phase CML do not suffer from an increase in bacterial or fungal infections until the advanced state of blastic crisis [21]. 

Regarding all gastrointestinal side effects (nausea, vomiting, stomatitis, and diarrhea), they were seen in 86% of patients, but they were more likely to be of grade 1 or 2 and did not force the patients to stop using the drug, except for stomatitis of grades 3 and 4, which occurred just in 1.5% of cases. Nausea due to direct irritant effect of the drug on the gastric mucosa was experienced by 32.5% of patients, which was not significantly different between males and females. Less frequently, vomiting was encountered in 13.5%, while stomatitis was of grades 1 and 2 in most occasions, associated with neutropenia, and was registered in 17.5% of patients, mainly in females. 

Druker et al. stated that nausea and vomiting was experienced by 42.5% and 14.7%, respectively [19]; however, these side effects could be reduced by taking imatinib with food, dividing the dose, or using antiemetic medications [21]. 

Elevation of the total serum bilirubin and other liver enzymes (ALT and AST) occurred in 2.5% and 1% of cases, respectively. This elevation occurred mostly in those patients with long durations of imatinib therapy rather than those with less than 1 year of treatment. The cause of this increase in bilirubin may the drug interaction that metabolized in the liver due to drugs like acetaminophen, or, in one patient, antituberculosis medication [22]. 

Hepatotoxicity is uncommon, occurring in approximately 3% of patients, usually within 6 months of the onset of imatinib use. Acute liver failure has been described [22]. Other causes of liver dysfunction should be excluded, including viral studies and examination of serum ferritin level, α1-anti-trypsin level, and concurrent use of hepatotoxic drugs such as acetaminophen. Imatinib is metabolized by the CYP3A4/5 P-450 enzyme system. Thus, caution needs to be taken when using drugs that are metabolized by the liver [22]. 

Grade 1 and 2 elevations of creatinine were noticed in 2.5% of cases, while proteinuria and hematuria were seen in 11% and 5.5% of cases, respectively. This may be related to the direct effect of the drug on the kidneys, or to underlying kidney diseases caused by diabetes or chronic uncontrolled hypertension. It was not significantly related to the use of other medications. 

During this study, discoloration of patients’ hair occurred in 9.5% of cases, in which the hair color changed from white to a dark color, while hair loss occurred in only 7% of CML patients. Hair depigmentation and hypopigmentation of the skin, probably related to the inhibition of the KIT receptor tyrosine kinase by imatinib, were reported in a French study, in which 133 patients with CML were treated with imatinib mesylate. Among these 133 patients, 5 men and 4 women (median age, 63.4 years; range, 53 to 75) with gray hair before treatment had progressive repigmentation of the hair (on the head in 8 patients and on the body and head in 1) during treatment. The median time between the end of interferon-alpha therapy and the start of treatment with imatinib mesylate was 5.7 months (range, 0.5 to 42). Hair repigmentation occurred after a median of 5 months (range, 2 to 14) of treatment with imatinib mesylate. How imatinib mesylate might induce hair repigmentation is a mystery [23]. Functional assays show inhibition of the DDR1 gene by imatinib mesylate, a potent inhibitor of BCR-ABL tyrosine kinase and c-kit tyrosine kinase. Interestingly, the use of imatinib can lead to vitiligo-like lesions, possibly due to inhibition of tyrosinase activity through the c-kit pathway blockade. The DDR1 gene is located between the HLA-E and HLA-C genes at chromosomal region 6p21, previously linked to vitiligo susceptibility in a Chinese population. In another study, imatinib has been proposed as a therapy for vitiligo because of its effects on the DDR1 gene [24,25]. 

Out of 200 patients in this study, weight gain was more significantly common among males than females (34% versus 22%); this was mostly because of fluid retention, which was statistically not significant. However, it is clear that fluid retention alone cannot account for the progressive increases in weight seen in some patients, as increased appetite has been reported by some patients while taking imatinib, which abates with discontinuation of treatment for any reason. Another aspect of weight gain has been observed with return of a normal appetite following the discontinuation of interferon-alpha treatment. Patients prone to weight problems need to be cautioned about the association of imatinib with weight gain [26]. 

In conclusion, imatinib mesylate is a well-tolerated drug, in our study and all undesirable effects were easy to manage. The most common hematologic side effect was anemia. Regarding the non-hematologic side effects, edema, bone pain, and nausea were the most commonly encountered conditions.

## CONFLICT OF INTEREST STATEMENT

The authors of this paper have no conflicts of interest, including specific financial interests, relationships, and/ or affiliations relevant to the subject matter or materials included.

## Figures and Tables

**Table 1:**
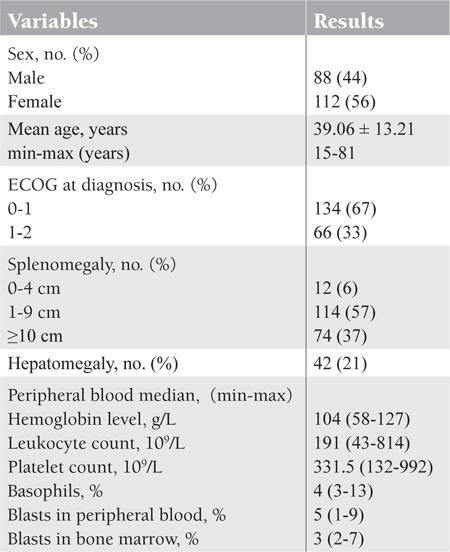
Pretreatment characteristics of 200 patients with CML

**Table 2:**
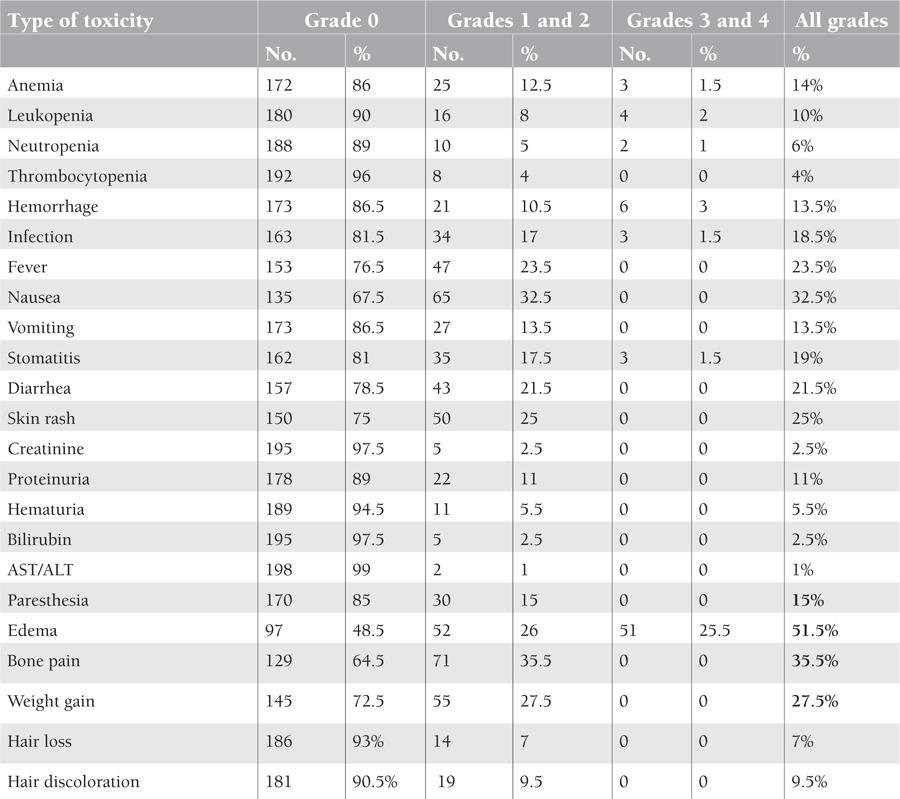
Distribution of the common toxicity effects according to grade. Significant results are given in bold

**Table 3:**
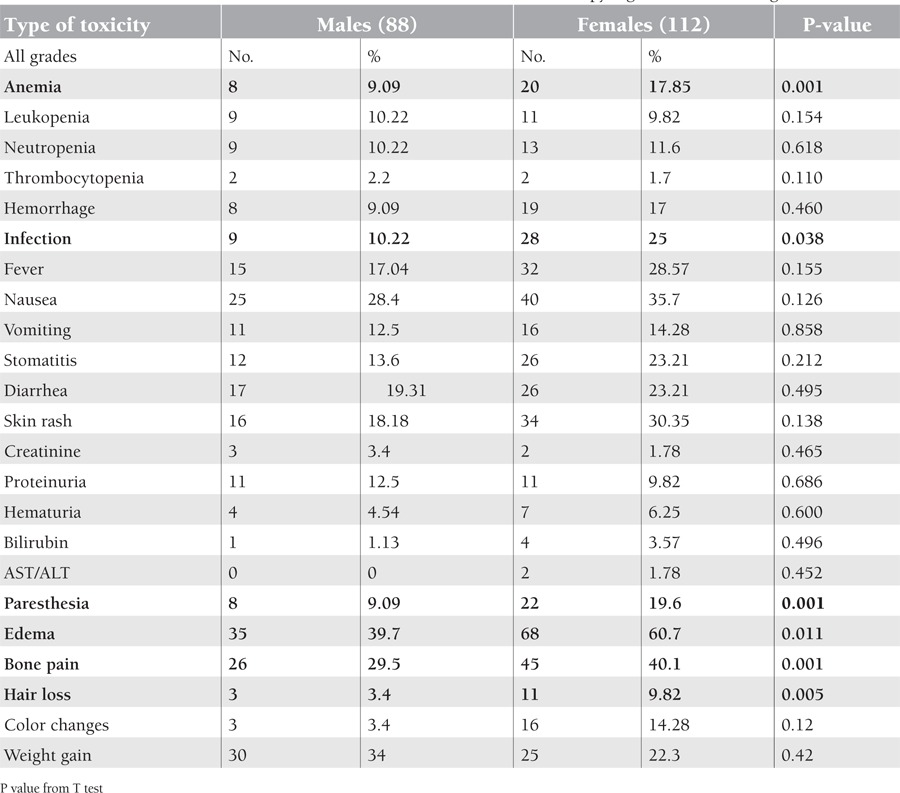
Difference in adverse effects between males and females on imatinib therapy. Significant results are given in bold
